# Evolutionary morphology of haptoral anchors in monogenoids (Dactylogyridae) of marine catfish (Siluriformes: Ariidae) from the Atlantic coast of South America

**DOI:** 10.1017/S0031182024000192

**Published:** 2024-04

**Authors:** Geusivam Barbosa Soares, Edson Aparecido Adriano, Marcus Vinicius Domingues, Abril Rodríguez-González, Juan Antonio Balbuena

**Affiliations:** 1 Departamento de Biologia Animal, Instituto de Biologia, Universidade Estadual de Campinas (UNICAMP), Campinas, São Paulo, Brazil; 2 Departamento de Ecologia e Biologia Evolutiva, Universidade Federal de São Paulo (UNIFESP), Diadema, São Paulo, Brazil; 3 Instituto de Estudos Costeiros, Universidade Federal do Pará (UFPA), Bragança, Pará, Brazil; 4 Universidad Nacional Autónoma de México (UNAM), Instituto de Biología, Laboratorio de Helmintología, Ciudad de México, México; 5 Institut Cavanilles de Biodiversitat i Biologia Evolutiva, Universitat de València, Valencia, Spain

**Keywords:** geometric morphometrics, molecular phylogeny, monogenoidea, morphological integration, phylomorphospace, shape and size variation

## Abstract

Exploring the phylogenetic signal of morphological traits using geometric morphometry represents a powerful approach to assess the relative weights of convergence and shared evolutionary history in shaping species' forms. We evaluated the phylogenetic signal in shape and size of ventral and dorsal haptoral anchors of 10 species of monogenoids (*Hamatopeduncularia*, *Chauhanellus* and *Susanlimocotyle*) occurring in marine catfish (Siluriformes: Ariidae) from the Atlantic coast of South America. The phylogenetic relationships among these species were mapped onto the morphospaces of shape and size of dorsal and ventral anchors. Two different tests (squared change-parsimony and *K*_mult_) were applied to establish whether the spatial positions in the phylomorphospace were influenced by phylogenetic relationships. A significant phylogenetic signal was found between anchor form and parasite phylogeny. Allometric effects on anchor shape were non-significant. Phylogenetically distant species on the same host differed markedly in anchor morphology, suggesting little influence of host species on anchor form. A significantly higher level of shape variation among ventral anchors was also found, suggesting that the evolutionary forces shaping ventral anchor morphology may operate with differing intensities or exhibit distinct mechanisms compared to their dorsal counterparts. Our results suggest that phylogenetic relationships were a key driver of changes in shape (but not size) of anchors of monogenoids of South American ariids. However, it seems that the emergence of the digitiform haptor in *Hamatopenducularia* and in some species of *Chauhanellus* played an important role in the reduction in anchor size and may cause secondary losses of anchors in other groups of monogenoids.

## Graphical abstract

**Figure:**
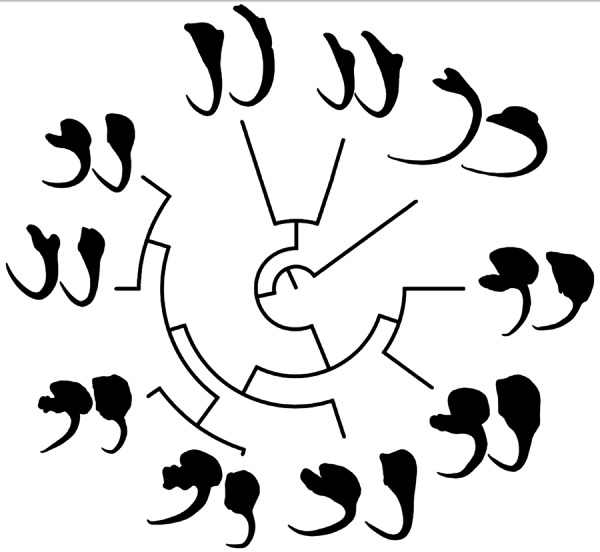

## Introduction

Monogenoidea Bychowsky, 1937 (Platyhelminthes) are primarily ectoparasites of fish (Whittington, 2005). These parasites have as a major taxonomic structure, the haptor, which plays a key role for attachment to the gills or body surface of the host (Bychowsky, 1957; Boeger and Vianna, 2006). This structure integrates sclerotized hard parts such as hooks, anchors and clamps or a combination of these elements. Importantly, many monogenoids exhibit high host specificity (Whittington *et al*., 2000), which indicates a highly specific adaptation to parasitize particular fish species.

Monogenoidea has demonstrated to be an excellent model system for studying the evolutionary processes that have driven parasite diversification and diversity (Poulin, 2002). Different studies have used these parasites to investigate the processes leading to their diversification and speciation (Šimková *et al*., 2002; Vanhove and Huyse, 2015), to elucidate the evolutionary association of hosts and parasites (Desdevises *et al*., 2002; Šimková *et al*., 2006; Šimková and Morand, 2008; Mendlová and Šimková, 2014; Vanhove *et al*., 2015; Míguez-Lozano *et al*., 2017; Rahmouni *et al*., 2022; Seidlová *et al*., 2022; Soares *et al*., 2023*a*) and to explore the relationship between phenotype variation in attachment organs and factors such as phylogeny and host specificity (Vignon *et al*., 2011; Sarabeev and Desdevises, 2014; Llopis-Belenguer *et al*., 2015; Khang *et al*., 2016; Rodríguez-González *et al*., 2017).

Some of these studies have been based on linear measurements of haptoral elements (e.g. Mladineo *et al*., 2013; Kmentová *et al*., 2020; Cruz-Laufer *et al*., 2022). This approach can leverage published datasets, enabling the analysis of extensive data volumes (Cruz-Laufer *et al*., 2022). However, a major limitation of linear measure-based morphometrics is the inherent fusion of size and shape information, leading to difficulties in disentangling these 2 aspects (Adams *et al*., 2004). Geometric morphometrics addresses this issue effectively and additionally provides visualization tools, such as shape deformation grids, that facilitate the interpretation and communication of intricate shape changes (Adams *et al*., 2013). Another advantage of geometric morphometrics is that shape analysis relies on homologous landmarks, ensuring that comparisons between individuals or taxa are based on corresponding anatomical points. While identification of landmarks is more time-consuming due to the relatively involved collection and processing of data, traditional morphometrics often rely on arbitrary or poorly defined measurements that make it difficult to assess homology (Adams *et al*., 2004).

Geometric morphometrics have been successfully utilized to investigate the evolutionary processes that have shaped the diversification of a wide range of organisms, including plants (Liu *et al*., 2015), fishes (Friedman *et al*., 2019), mites (Kerschbaumer and Pfingstl, 2021) and parasites (Vignon *et al*., 2011; Baillie *et al*., 2019; Soares *et al*., 2023a). In Monogenoidea, different studies, using geometric morphometric data of the haptoral anchors, associated with molecular phylogenies have underscored the usefulness of this approach in tackling diverse evolutionary inquiries (Llopis-Belenguer *et al*., 2015; Khang *et al*., 2016; Rodríguez-González *et al*., 2017; Rahmouni *et al*., 2021; Soares *et al*., 2023a).

In the present study, we integrate geometric morphometrics of haptoral anchors and DNA sequences in a comparative phylogenetic context, in order to investigate the evolution of form (i.e. the combination of shape and size *sensu* Klingenberg, 2016) of 10 species from 3 monogenoid genera (*Hamatopeduncularia* Yamaguti, 1953, *Chauhanellus* Bychowsky & Nagibina, 1969 and *Susanlimocotyle* Soares, Domingues and Adriano, 2021) that parasitize Ariidae (Siluriformes) from South America. Haptoral anchors were chosen for analysis because they are not subjected to large variation due to contraction or flattening on fixation (Vignon, 2011) and are crucial for effective attachment to the host. In fact, Šimková *et al*. (2002) indicate that the morphology of the haptor is, to a large degree, determined by adaptation to the host and to attachment to specific sites within their hosts, which has been demonstrated in, for instance, *Lamellodiscus* spp. (Poisot *et al*., 2011). Thus, similarity in anchor morphology could result from homoplasy, indicating convergent evolution. However, shared evolutionary history can also play a major role in determining anchor shape, as shown in *Ligophorus* spp. (Rodríguez-González, 2017). So, anchor morphology is probably shaped by a complex interplay between adaptive forces and phylogenetic constraints, the effects of which may vary among different monogenoids (Messu Mandeng *et al*., 2015; Rodríguez-González *et al*., 2016, 2017).

Furthermore, research indicates that the intensity and interaction of adaptive forces and phylogenetic constraints can manifest differently in various haptoral elements (Vignon *et al*., 2011; Rodríguez-González *et al*., 2015). For example, in *Ligophorus cephali* Rubtsova, Balbuena, Sarabeev, Blasco–Costa & Euzet, 2006 on *Mugil cephalus* (Linnaeus, 1758), a greater control has been observed over the shape and size of the ventral pair of anchors compared to their dorsal counterparts. This difference is noteworthy as the ventral anchors seem responsible for a firmer attachment to the gills (Llopis-Belenguer *et al*., 2015; Rodríguez-González *et al*., 2015). In fact, evidence indicates that ventral and dorsal anchors in species of *Ligophorus* and *Cichlidogyrus* exhibit relatively independent evolutionary trajectories, mirroring the functional distinction in their attachment roles (Vignon *et al*., 2011; Rodríguez-González *et al*., 2015).

The objectives of the present study were (1) to assess the relative influences of convergence and shared evolutionary history on anchor form on the dactylogyrid parasites of South American ariids, (2) assess shape and size differences between ventral and dorsal anchors that might provide cues for different functional attachment roles and (3) to use anchor morphology to understand the relationships and evolutionary history of the 3 monogenoid genera studied. Thus, special attention was given to examine whether the morphology of the anchors serves as a basis for synonymizing *Hamatopeduncularia* and *Chauhanellus*, as suggested previously (Kearn and Whittington, 1994; Lim, 1994, 1996). Furthermore, considering the recently suggested ancestral relationship between *Susanlimocotyle* and the latter 2 genera (Soares *et al*., 2021, 2023*b*), we also assessed whether there are patterns in the evolutionary changes of anchor morphology within these monogenoids.

## Materials and methods

### Study area, host and parasite samples

The species of fish and parasites from 4 localities in the Brazilian coast (Table 1) are the same used in our previous studies (Soares *et al*., 2023*a*, 2023*b*). The morphological analysis of parasites includes data of all species of *Hamatopeduncularia*, *Chauhanellus* and *Susanlimocotyle* occurring on ariid catfish in the Brazilian coast (10 species in total) (Table 1). A previously published phylogenetic tree based of concatenated partial sequences of genes 18S rDNA, ITS1, 5.8S rDNA and ITS2 performed using Bayesian inference (Soares *et al*., 2023*a*) was used to assess the relationships between morphology and evolutionary history of the monogenoid species. The choice of these molecular markers is justified by the relatively large number of sequences available for different species of monogenoid of South American ariid fishes.

**Table 1.** Host species, locality (geographical coordinates) and associated species of *Chauhanellus*, *Hamatopeduncularia* and *Susanlimocotyle* used in the present study

Host	Locality	Parasite
*Amphiarius rugispinis* (Valenciennes, 1840)	Ajuruteua (0°49′31″N; 46°36′29″W), Bragança, PA, Br	*C. hamatopeduncularoideum* Domingues, Soares and Watanabe, 2016, OP681531^a^ *C. neotropicalis* Domingues and Fehlauer, 2006
*Aspistor luniscutis* (Valenciennes, 1840)	Cananéia (25°02′09.2″S; 47°54′57.8″W), SP, Br	*C. neotropicalis* OP681530^a^ *H. cangatae* Domingues, Soares and Watanabe, 2016, OP681532^a^
*Aspistor quadriscutis* (Valenciennes, 1840)	Ajuruteua (0°49′31″N; 46°36′29″W), Bragança, PA, Br	*C. neotropicalis* *H. cangatae*
*Bagre bagre* (Linnaeus, 1766)	Ajuruteua (0°49′31″N; 46°36′29″W), Bragança, PA, Br	*H. bagre* Hargis, 1955, OP681526^a^
*Genidens barbus* (Lacepède, 1803)	Cananéia (25°02′09.2″S; 47°54′57.8″W), SP, Br	*C. boegeri* Domingues and Fehlauer, 2006, OP681529^a^
	Estuary of Patos Lagoon (32°08′05.7″S; 52°06′11.2″W), RS, Br	*C. boegeri* *C. riograndinensis* Soares, Martins, Vianna, Domingues and Adriano, 2023*b*, OP681534^a^
*Genidens genidens* (Cuvier, 1829)	Estuary of Patos Lagoon (32°08′05.7″S; 52°06′11.2″W), RS, Br	*C. boegeri* *C. riograndinensis*
*Notarius grandicassis* (Valenciennes, 1840)		*C. neotropicalis* *H. cangatae*
*Sciades couma* (Valenciennes, 1840)	Caratateua (1°59′41.91″S; 46°43′21.385″W), Bragança, PA, Br	*C. hamatopeduncularoideum* *C. velum* Domingues, Soares and Watanabe, 2016 *C. boegeri*
*Sciades herzbergii* (Bloch, 1794)	Ajuruteua (0°49′31″N; 46°36′29″W), Bragança, PA, Br	*C. boegeri* *C. susamlimae* Domingues, Soares and Watanabe, 2016 *C. velum* OP681528^a^ *S. narina* Soares, Domingues and Adriano, 2021, OP681525^a^
*Sciades passany* (Valenciennes, 1840)	Caratateua (1°59′41.91″S; 46°43′21.385″W), Bragança, PA, Br	*C. neotropicalis* *C. susamlimae* OP681527^a^ *C. velum*
*Sciades proops* (Valenciennes, 1840)	Ajuruteua (0°49′31″N; 46°36′29″W), Bragança, PA, Br	*C. hypenocleithrum* Domingues, Soares and Watanabe, 2016, OP681533^a^

PA, Pará; SP, São Paulo; RS, Rio Grande do Sul; BR, Brazil.

a GenBank accession numbers of the DNA sequences of genes *18S rDNA*, *ITS1*, *5.8S rDNA* and *ITS2* used for the phylogenetic reconstruction of the parasites by Soares *et al*. (2023*a*). *C*. = *Chauhanellus*; *H.* = *Hamatopeduncularia*; *S.* = *Susanlimocotyle.*

### Morphometric data

In landmark-based geometric morphometrics, anatomical landmarks (LMs) are identified and digitized on images of the biological structure under study (Klingenberg, 2010). Herein we placed LMs on the haptoral anchors of the monogenoids following Rodríguez-González *et al*. (2017). The haptor of the species studied includes 2 pairs of ventral and dorsal anchors (VA and DA, respectively) (see Fig. 2 in Soares *et al*. (2023*a*)). We conducted parallel analyses of VA and DA since potential differences between them may provide insights into the distinct selective pressures influencing their morphology due to putative differing roles in attachment (Rodríguez-González *et al*., 2017). Drawings of VA and DA were taken from the original descriptions of the parasites (holotype) (Domingues and Fehlauer, 2006; Domingues *et al*., 2016; Soares *et al*., 2021, 2023*b*) and were used to place the LMs. One VA and 1 DA of each monogenoid species were processed independently. In each anchor, 5 homologous LMs were placed as per Soares *et al*. (2023*a*). To capture anchor morphology more accurately, semilandmarks (SLMs) were inserted between each LM (Mitteroecker and Gunz, 2009; Llopis-Belenguer *et al*., 2015; Rodríguez-González *et al*., 2015), following the methods for sliding the SLMs (Bookstein *et al*., 2002). Five groups of 6–29 SLMs were placed equidistantly between LM pairs (for descriptions and locations of LMs and SLMs, see Soares *et al*. (2023*a*)). The morphology of VA and DA was defined by the Cartesian coordinates (*x*, *y*) of the 83 anatomical points (i.e. LMs and SLMs).

Digitalization of the LMs and SLMs was processed with the TpsDig v2.32 (Rohlf, 2022). Generalized Procrustes analysis in MorphoJ v1.07a (Klingenberg, 2011) was employed to obtain matrices of shape coordinates of VA and DA (datasets 1 and 2, respectively). This analysis removes all information related to position, scale and orientation. Centroid size (CS), estimated as the summed squared distances of each LM from the centroid of the form (Zelditch *et al*., 2012), was used as a measure of anchor size. To visualize interspecific variation in anchor shape, we subjected the matrices of generalized Procrustes analysis coordinates of the VA and DA to principal component analysis (PCA) based on the covariation matrix.

### Assessing the influence of size on anchor shape

We assessed the effects of size on shape variation of the anchors (i.e. interspecific allometry) independently for VA and DA by means of a multivariate regression analysis (Klingenberg, 2016). We used the Procrustes shape coordinates of VA and DA and their log-transformed centroid size (logCS) as input in a multivariate regression through the origin (Lim and Gibson, 2009; Klingenberg *et al*., 2012). Then, we mapped the residuals from this regression onto the phylogenetic tree of the monogenoids. A sizeable variation between the original datasets and the residuals would suggest that evolutionary allometry (i.e. the allometry between traits measured across species) played an important role in anchor evolution in these monogenoids.

To avoid incorrect interpretations due to a violation of the assumption of independent sampling (Harvey and Pagel, 1991), we also assessed the effect of size on anchor shape with the phylogenetic independent contrast (PIC) correction (Felsenstein, 1985). Since no evidence for allometry to the PIC-corrected analyses was found (*P* > 0.07 in VA and DA), the effect of evolutionary allometry was not further considered.

### Evaluating phylogenetic signal in anchor shape and size

To test whether closely related monogenoids tend to have more similar anchors to each other than of more distantly related monogenoids, we evaluated phylogenetic signal in anchor shape and size. For that, we mapped a topology of the phylogenetic tree of our 10 monogenoid species onto the morphospace defined by the 2 first PCA scores (PC1 and PC2) of shapes and onto logCS (anchor size) using squared change-parsimony assuming a model of Brownian-motion (BM) evolution (Klingenberg and Marugán-Lobón, 2013). Phylogenetic signal was evaluated with MorphoJ. Its significance (*P* < 0.05) was established by a permutation test in which the topology was held constant and the principal component scores for each taxon were randomly permuted 10 000 times across the tree (Maddison, 1991; Klingenberg and Gidaszewski, 2010). If there were no correlation between phylogeny and morphometric data, the tree length value should be small (closer to 0 than to 1) and non-significant. Given the disagreement on which approach is more appropriate to measure the phylogenetic signal (Blomberg *et al*., 2003; Adams, 2014), we also used *K*_mult_ (generalization of Blomberg's *K*) (Adams and Otarola-Castillo, 2013; Adams, 2014) to test for the phylogenetic signal in our data. *K*_mult_ quantifies the extent to which a trait displays phylogenetic signal following BM evolution (Diniz-Filho *et al*., 2012). *K*_mult_ = 0 suggests no phylogenetic signal, *K*_mult_ = 1 indicates that the trait distribution perfectly conforms to BM, *K*_mult_ < 1 correspond to trait variation that is larger than expected between taxa of the same lineage and *K*_mult_ > 1 indicates stronger similarities among closely related species than expected under BM. The significance of *K*_mult_ (*P* < 0.05) was established based on comparison of the observed value with those obtained in 999 randomizations (Liu *et al*., 2015). The tests were performed with function *physignal* of the geomorph package v3.0.1 (Adams, 2014) in R v4.1.0 (R Core Team, 2022).

## Results

### Phylogenetic signal in anchor shape and anchor size

The PCA performed with the covariance matrix of LM data of both VA and DA shows that a large part of the variation is contained in relatively few dimensions. The first 2 principal components accounted for 78.5 and 78.3% of the total shape variation in VA and DA, respectively (Table 2). Eigenvalues and variance explained by each principal component are given in Supplementary Table S1.

**Table 2.** PCA of variation among the shapes of species for ventral and dorsal anchors of monogenoids from ariids

Anchor	Eigenvalue	Total variance (%)
Ventral		
PC1	2.68×10^−2^	49.8
PC2	1.54×10^−2^	28.7
Dorsal		
PC1	2.27×10^−2^	58.6
PC2	7.64×10^−3^	19.7

**Figure 1.**
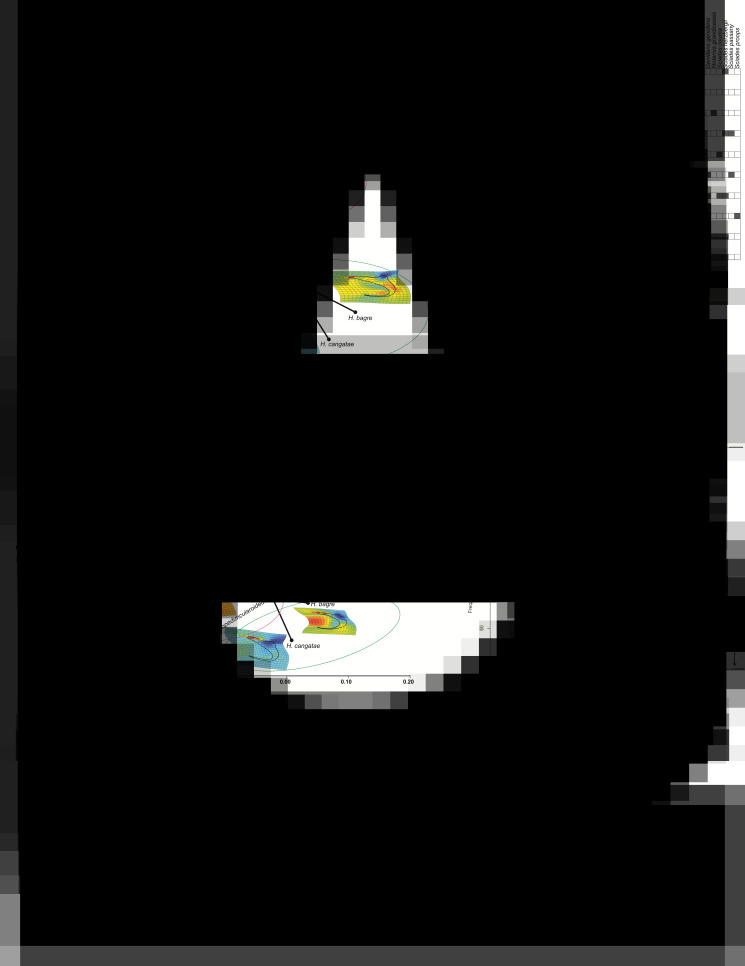
Phylomorphospace showing changes in the shape of ventral (a) and (b) dorsal haptoral anchors of the species of monogenoids (*Susanlimocotyle*, *Hamatopeduncularia* and *Chauhanellus* spp.) studied mapped onto phylogeny. Estimated changes in anchor shapes are shown as thin-plate-spline deformation grids with colour-scaled coded Jacobian expansion factors (red for factors >1, indicating expansion; strong blue for factors between 0 and 1, indicating contraction) were used. The insert shows the parts of an anchor in monogenoids species from ariids. *S.*, *Susanlimocotyle*; *H.*, *Hamatopeduncularia*; *C*., *Chauhanellus*; The coloured circles represent the clusters formed. (c) Bayesian tree based on partial sequences of genes 18S rDNA, ITS1, 5.8S rDNA and ITS2 sequences of representative individuals of 10 monogenoids species of the genera *Susanlimocotyle*, *Hamatopeduncularia* and *Chauhanellus* (posterior probabilities support values are given above the branches); and host–parasite distribution. (d–e) Histogram of *K*_mult_ values obtained from 999 permutations of the ventral (d) and (e) dorsal anchors shape data and the phylogeny, with the position of observed value of *K*_mult_ identified.

In the phylomorphospace (Fig. 1a and b), in which the phylogeny of monogenoids (Fig. 1c) was projected onto the morphospace defined by PC1 and PC2 of the VA and DA shape, congeneric species tended to cluster together. Only *Chauhanellus hamatopeduncularoideum* from *Amphiarius rugispinis* and *Sciades couma* did not follow this trend. By contrast, species reported parasitizing the same hosts (i.e. *Chauhanellus boegeri*, *C. velum* and *C. susamlimae* from *Sciades herzbergii*) did not group together in the phylomorphospace. Both approaches, MorphoJ (VA: tree length = 0.249, *P* = 0.007 and DA: tree length = 0.187, *P* = 0.001), and *K*_mult_ (VA: *K*_mult_ = 0.78, *P* = 0.01 and DA: *K*_mult_ = 1.1, *P* = 0.001) (Fig. 1d–e) supported a significant relationship signal between the shape of VA and DA, and phylogenetic position of the monogenoids.

The deformation grids of each species provide a visual representation of their deviation from the average anchor shape (VA and DA) of the species studied (see parts of anchors in insert in Fig. 1a and b). Based on this evidence, we identify 4 clusters: (i) *Chauhanellus* spp. except *C. hamatopeduncularoideum* (see Table 1 for *Chauhanellus* spp., and Fig. 1c for host–parasite distribution), characterized by a VA with truncated inner root, expanded outer root, short shaft and evenly curved to point; DA with poorly developed inner root, expanded outer root and wide base (Fig. 1a and b, cluster marked with a yellow circle); (ii) *Hamatopeduncularia* spp. (*H. bagre* from *Bagre bagre*; and *H. cangatae* from *A. luniscutis*, *A. quadriscuti* and *N. grandicassis*), characterized by a VA with long inner root, non-expanded outer root, long shaft, curved to point; DA with long inner root, non-differentiated outer root, narrow base (Fig. 1a and b, cluster marked with a green circle); (iii) the monotypic *Susanlimocotyle narina* (from *S. herzbergii*), characterized by a VA with developed inner and outer roots, long shaft, evenly curved to point (*Hamatopeduncularia* morphology); DA with developed inner root, outer root expanded and wide base (*Chauhanellus* morphology) (Fig. 1a and b, cluster marked with a red circle), with characteristics intermediate between cluster ii (at the VA) and between clusters i and ii (at the DA), which seem to represent the characteristics shared with the species of *Hamatopeduncularia* and *Chauhanellus*, consistent with the close relationship of these species suggested by the phylogenetic tree (Fig. 1c); and (iv) *C. hamatopeduncularoideum* (from *A. rugispinis* and *S. couma*), characterized by a VA with long inner root, not expanded outer root, long shaft, curved to point (*Hamatopeduncularia* morphology); DA with poorly developed inner root, expanded outer root and wide base (*Chauhanellus* morphology), with intermediate characteristics between cluster ii (at the VA) and between i (at the DA), and *C. hamatopeduncularoideum* covering the same swath in phylomorphospace for VA and DA (see Fig. 1a and b, cluster marked with a pink circle).

**Figure 2.**
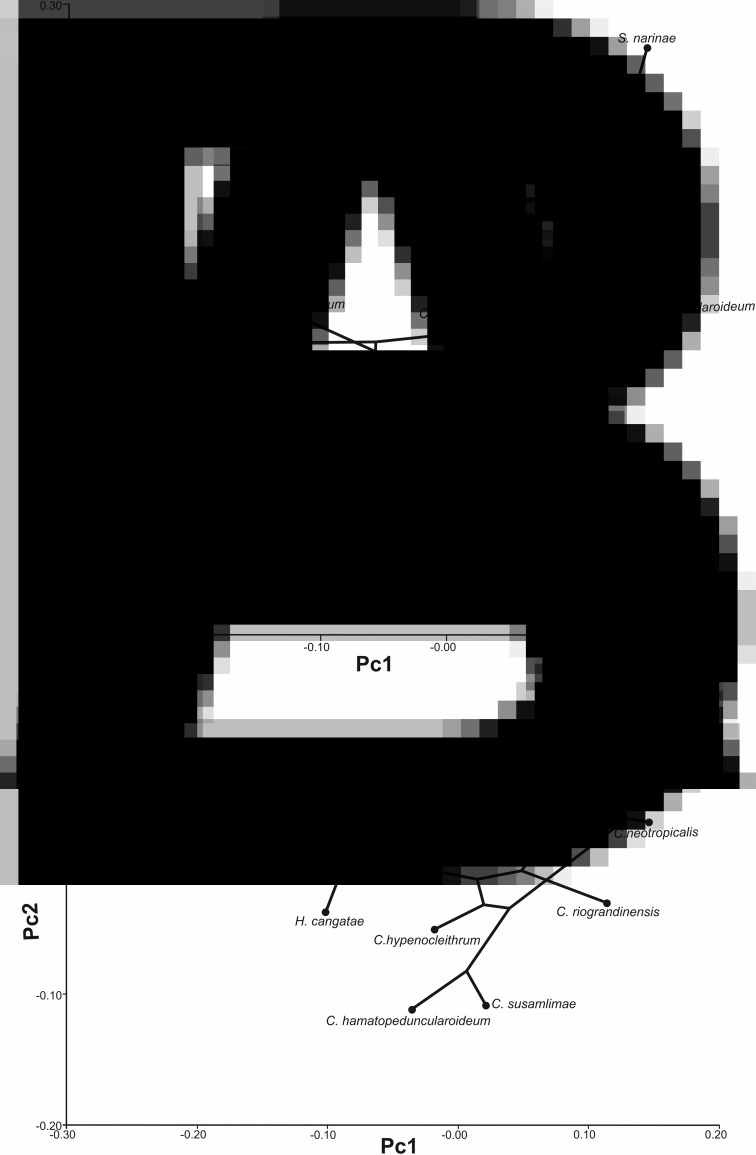
Phylomorphospace showing changes in shape (corrected for size) of ventral (a) and (b) dorsal haptoral anchors of the species of monogenoids (*Susanlimocotyle*, *Hamatopeduncularia* and *Chauhanellus* spp.) studied mapped onto phylogeny. Species abbreviations as in Fig. 1.

The phylogeny of monogenoids projected onto the morphospace defined by allometry-free (size-corrected) PC1 and PC2 of anchor shape yielded a tree length of 0.02 for VA and DA (Fig. 2a and b). The multivariate regression of Procrustes coordinates on logCS, provided evidence for an allometric relationship between shape and size only for DA (VA: *P* = 0.5; DA: *P* = 0.01), accounting for 29.7% of the total shape variation of DA. Phylogenetic signal was again highly significant, both in MorphoJ (*P* = 0.007 each) and *K*_mult_ (size-corrected) (VA: *K*_mult_ = 0.72, *P* = 0.009 and DA: *K*_mult_ = 0.98, *P* = 0.001). The scatter graph of VA (Fig. 2a) showed small branches of *C. susamlimae*, *C. velum*, *C. riograndinensis* and *H. bagre* than in the PCA uncorrected for size (Fig. 1a). By contrast, the branches of *C. boegeri*, *C. neotropicalis*, *C. susamlimae*, *C. velum* and *C. riograndinensis* were larger than the original PCA in the DA scatterplot (compare Fig. 1b with Fig. 2b). Whereas the position of species in the original and size-corrected was similar in the VA phylospaces, it was not the case in the DA phylospaces. Consequently, allometry had a significant effect on the overall variation of DA shape, but not on VA shape.

**Figure 3.**
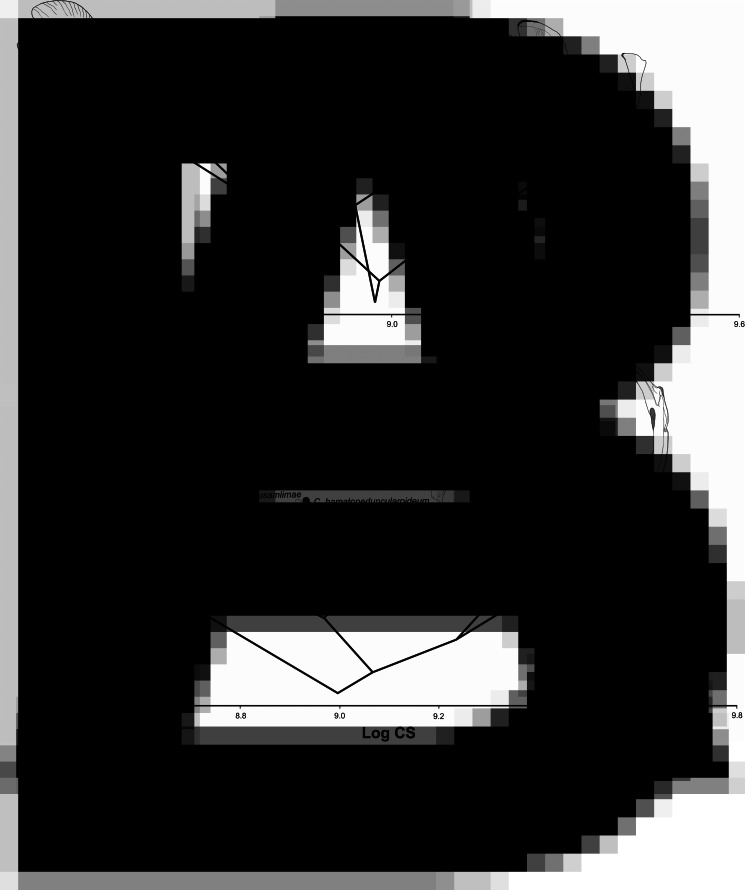
Projection of phylogenetic tree from monogenoids (*Susanlimocotyle*, *Hamatopeduncularia* and *Chauhanellus* spp.) onto log centroid size (logCS) of ventral (a) and dorsal (b) haptoral anchors. Species abbreviations as in Fig. 1. The anchors displayed are scaled as per the logCS scale to convey the gradient in size.

In Fig. 3, the molecular phylogeny projected onto the gradient in size (logCS) of VA and DA is shown along with the cumulative branch length from the root of the tree. This mapping resulted in tree lengths of VA and DA of 0.34 and 0.62, respectively, computed in units of logCS distance along all branches. Phylogenetic signal tested in MorphoJ by random permutation of logCS was not statistically significant in both anchors (VA: *P* = 0.07 and DA: *P* = 0.12). However, *K*_mult_ indicated a significant phylogenetic signal in VA but not in DA (VA: *K*_mult_ = 0.99, *P* = 0.03; DA: *K*_mult_ = 0.68, *P* = 0.11).

## Discussion

Monogenoids of the genera *Hamatopeduncularia*, *Chauhanellu*s and *Susanlimocotyle*, parasitic on South American ariids, exhibit distinct variations in anchor shape (Domingues *et al*., 2016; Soares *et al*., 2021, 2023*b*), and these differences are clearly reflected in their positions within the phylomorphospace (Fig. 1a and b). Thus, it is not surprising that for the 3 genetic lineages (*Susanlimocotyle*, *Hamatopeduncularia* and *Chauhanellus*) (Fig. 1c), the VA and DA shape exhibit a significant phylogenetic signal, suggesting that evolutionary history played an important role in determining the shape of haptoral anchors. This agrees with other studies, which suggest a consistent relationship between anchor morphology and phylogeny in monogenoids (Sarabeev and Desdevises, 2014; Khang *et al*., 2016; Rodríguez-González *et al*., 2017).

Visually, the distribution of anchors in phylomorphospace was more convincing for the VA than for DA, indicating the formation of clusters (Fig. 1a and b), especially regarding the separation of *Hamatopeduncularia* and *Chauhanellus* lineages into 2 distinct clusters. Interestingly, the *K*_mult_ corresponding to shape was <1 for VA and >1 for DA (Fig. 1d–e). Thus, the phenotypic variation in VA is greater than expected between taxa of the same lineage (Adams, 2014). This indicates that the evolutionary processes acting on VA shape did not act with similar intensity, or were not the same as in DA in these genera.

In addition, the deformation grids plotted in Fig. 1a indicate that the inner and outer roots of the VA are more differentiated than those of the DA counterparts in species of each genus (Fig. 1b). In general, the anchor roots of Dactylogyridae are the point of connection and articulation with the bars, aided by the insertions of the haptoral muscles. Functionally, they have the role of controlling the intensity of attachment to the host (Kearn, 1994). Thus, the differences observed between VA and DA can be explained in terms of different functional roles of these structures, which seems to be common in Dactylogyridae (Vignon *et al*., 2011; Llopis-Belenguer *et al*., 2015; Rodríguez-González *et al*., 2015).

Interestingly, the VA shape in *C. hamatopeduncularoideum*, characterized by an inner root long, outer root not expanded, long shaft, curved to point, exhibits a greater resemblance in the phylomorphospace to those found in *Hamatopeduncularia* species rather than other *Chauhanellus* species (Fig. 1a). One could speculate that similarity in the shape of the anchors may result from adaptation to the gill morphology of the host. However, *C. hamatopeduncularoideum* does not share any host with *Hamatopeduncularia* spp. (Fig. 1c). Alternatively, *C. hamatopeduncularoideum* could be considered a species of *Hamatopeducunlaria*. In fact, some authors (Kearn and Whittington, 1994; Lim, 1994, 1996; Lim *et al*., 2001; Domingues *et al*., 2016) have suggested that *Chauhanellus* and *Hamatopeduncularia* may be considered synonyms, because certain species within each genus share morphological characteristics that were originally used to differentiate the 2 genera in the past (Soares *et al*., 2021, 2023*b*). However, evidence from molecular data does not support neither the inclusion of *C. hamatopeduncularoideum* within *Hamatopeduncularia*, nor the synonymy of the 2 genera (Fig. 1c) (Soares *et al*., 2021, 2023*b*).

Alternatively, the presence of *Hamatopeduncularia*-like anchors in *Chauhanellus* species suggests that this morphology might have been present in the common ancestor of both genera (Kearn and Whittington, 1994). Thus, reversal to the ancestral character state would account for the anchor morphology in *C. hamatopeduncularoideum*, as suggested for other structures in some monogenoids (Šimková *et al*., 2006).

Šimková *et al*. (2006) carried out ancestral state reconstructions on *Dactylogyrus* species, revealing a shift in haptor anchor shapes from type 2 to type 1 for the majority of species (see Šimková *et al*., 2006, p. 1028, Table 1 for anchor shapes). However, the authors noted a reversion to the ancestral state in derivative species. This observation appears to be applicable to the *Hamatopeduncularia*-type anchors in *C. hamatopeduncularoideum.*

Indeed, the VA shape of *S. narinae* also conforms to that of *Hamatopeduncularia* (see parts of anchors in insert in Fig. 1a), whereas the DA shape seems a composite of those of *Hamatopeduncularia* and *Chauhanellus* (see parts of anchors in insert in Fig. 1b). This suggests that some character states of *Susanlimocotyle* shared *Hamatopeduncularia* and *Chauhanellus* might represent evolutionary ancestral forms (Fig. 1a–c).

An additional feature of *C. hamatopeduncularoideum* shared with *Hamatopeduncularia* spp. is the presence of a digitiform haptor. This character was originally considered as diagnostic of *Hamatopeduncularia* (Yamaguti, 1953), but it was later found in some *Chauhanellus* spp. (e.g. *C. susamlimae* and *C. riograndinensis*) (Kearn and Whittington, 1994; Lim, 1994, 1996; Lim *et al*., 2001; Domingues *et al*., 2016; Soares *et al*., 2023*b*). Interestingly, the *Chauhanellus* species that have a digitiform haptor (*C. hamatopeduncularoideum*, *C. susamlimae* and *C. riograndinensis*) have smaller anchors than closely related congeneric species (Fig. 3). This could account for the lack of significant phylogentic signal in anchor size found in most analyses. Only the *K*_mult_ result for DA was significant, but Fig. 3b suggests that the relationship between size and phylogenetic relatedness is not strong.

Kearn and Whittington (1994) suggested that an important innovation in some ancestral ancyrocephalines (Dactylogyridae) was the development of hooklet-bearing papillae (=digitiform haptor) with the ability to elongate. According to these authors, this provided these parasites with multiple attachment points to the gills, and offered little resistance, minimizing the threats of displacement by water currents, allowing more versatility in attachment sites. Thus, the digitiform haptor decreased the function of the anchors, which resulted in a reduction of their size (Kearn and Whittington, 1994). This scenario is supported by the present study, as the presence of a digitiform haptor in *C. hamatopeduncularoideum*, *C. susamlimae* and *C. riograndinensis* coincides with a reduction in anchor size. Likewise, the digitiform haptor may have caused the secondary loss of anchors in other dactylogirid genera (e.g. *Trinigyrus* Hanek, Molnar & Fernando (1974) sensu Kritsky *et al*. (1986)). However, in other genera with no digitiform haptor, like *Dactylogyrus* and *Dogielius*, the ventral bar is significantly reduced and ventral anchors are altogether absent (Pravdová *et al*., 2018). Thus, other selective forces would account for the reduction of haptors elements. In any case, this observation strengthens the proposition that dorsal and ventral anchors may undergo distinct evolutionary trajectories within the Dactylogyridae.

## Conclusion

Our study suggests that phylogeny has driven the evolution of shape but not size of the anchors of monogenoids from South American ariids. However, it seems that the emergence of the digitiform haptor in *Hamatopenducularia* and some species of *Chauhanellus* played an important role in the reduction of anchors, as suggested by other authors, and may account for secondary losses in other groups of monogenoids.

Nevertheless, we acknowledge the limited scope of our study. While typical geometric morphometric studies analyse several specimens per species, our investigation employed a single representative per species due to lack of specimen availability. Our approach rest on the assumption that intraspecific variation and measurement error are smaller than interspecific differences (Klingenberg and Marugán-Lobón, 2013). This premise appears substantiated given that morphological differences in both size and shape of the anchors between species were clear and substantial (Fig. 3). Nevertheless, we must acknowledge the potential impact of even minor levels of intraspecific variation or measurement error on the conclusions drawn from our study. Hence, while our findings offer valuable insights into the phylogenetic effects on anchor form, future research incorporating multiple specimens will undoubtedly contribute to a more nuanced picture of the evolution of anchor morphology of monogenoids of ariid fishes.

In addition, future studies should also use molecular markers from different regions (i.e. 28S rDNA, COI) and include a wider range of taxa, including the type species of each genus (*Hamatopeduncularia arii* Yamaguti, 1953 and *Chauhanellus oculatus* Bychowsky & Nagibina, 1969) and representatives of New-World and Old-World lineages as proposed by Soares *et al*. (2023*b*).

## Supporting information

Soares et al. supplementary material Soares et al. supplementary material

## Data Availability

Datasets 1–2 required to perform all the analyses are deposited on Zenodo (https://zenodo.org/records/10412631).

## Supplementary material

The supplementary material for this article can be found at https://doi.org/10.1017/S0031182024000192
