# Supplementary material for: Evolutionary morphology of haptoral anchors in monogenoids (Dactylogyridae) of marine catfish (Siluriformes: Ariidae) from the Atlantic coast of South America
Source: Parasitology. 2024 Feb 23;151(4):390–9. doi: 10.1017/S0031182024000192 (PMC11044069; doi:10.1017/S0031182024000192)
Supplement: Soares et al. supplementary material [file S0031182024000192sup001.pdf]

Supplementary table S1. Eigenvalues and percentage of variance explained by each principal component for ventral and dorsal anchors

**Ventral anchors**	Eigenvalues	% Variance	Cumulative %
**PC 1**	0.02682284	49.802	49.802
**PC 2**	0.01544668	28.680	78.482
**PC 3**	0.00638345	11.852	90.335
**PC 4**	0.00259704	4.822	95.156
**PC 5**	0.00105603	1.961	97.117
**PC 6**	0.00075425	1.400	98.518
**PC 7**	0.00051746	0.961	99.478
**PC 8**	0.00014575	0.271	99.749
**PC 9**	0.00013515	0.251	100.000

**Dorsal anchors**	Eigenvalues	% Variance	Cumulative %
**PC 1**	0.02275024	58.642	58.642
**PC 2**	0.00763765	19.687	78.329
**PC 3**	0.00507226	13.074	91.403
**PC 4**	0.00191043	4.924	96.328
**PC 5**	0.00055055	1.419	97.747
**PC 6**	0.00032501	0.838	98.585
**PC 7**	0.00024599	0.634	99.219
**PC 8**	0.00019878	0.512	99.731
**PC 9**	0.00010430	0.269	100.000
